# Branched-Chain Amino Acids and Testosterone in Older Adults With Type 2 Diabetes: Cross-Sectional and Longitudinal Findings From a Post Hoc Analysis of an Amino Acid Supplementation Trial

**DOI:** 10.7759/cureus.92180

**Published:** 2025-09-12

**Authors:** Erika Matsuda, Takaaki Matsuda, Kosuke Kojo, Yoshinori Osaki, Yasuhiro Suzuki, Hiroaki Suzuki, Bryan Mathis, Motohiro Sekiya, Hiroyuki Nishiyama, Hitoshi Shimano

**Affiliations:** 1 Department of Endocrinology and Metabolism, Institute of Medicine, University of Tsukuba, Tsukuba, JPN; 2 Department of Endocrinology and Metabolism, University of Tsukuba Hospital, Tsukuba, JPN; 3 Tsukuba Clinical Research and Development Organization (T-CReDO), University of Tsukuba, Tsukuba, JPN; 4 Department of Urology, University of Tsukuba Hospital, Tsukuba, JPN; 5 Institute of Systems and Information Engineering, University of Tsukuba, Tsukuba, JPN; 6 Department of Food and Health Sciences, Faculty of Human Life Sciences, Jissen Women's University, Tsukuba, JPN; 7 Department of Cardiovascular Surgery, Institute of Medicine, University of Tsukuba, Tsukuba, JPN; 8 Department of Urology, Institute of Medicine, University of Tsukuba, Tsukuba, JPN

**Keywords:** aged adult, anabolic agents, body composition, branched-chain amino acids (bcaas), insulin resistance, postmenopause, sex hormone-binding globulin, testosterone (tt), type 2 diabetes mellitus (dm)

## Abstract

Introduction: Branched-chain amino acids (BCAAs) and sex hormones share anabolic effects on skeletal muscle. As cross-sectional associations in older adults with type 2 diabetes (T2D) remain unclear, we analyzed potential causal effects of BCAA supplementation in this population.

Methods: This was an exploratory, post hoc analysis of a 24-week amino acid supplementation trial in older adults with T2D (BCAA 8 g or soy protein 7.5 g). Thirty-five participants (22 men, 13 women) were included. Total testosterone (TT), free testosterone (FT), and sex hormone-binding globulin (SHBG) were measured. Spearman’s rank correlation assessed cross-sectional associations, and paired t-tests or Wilcoxon signed-rank tests analyzed pre- and post-supplementation changes.

Results: At baseline, men showed a negative correlation between serum BCAA and TT (ρ = -0.486) but not FT, whereas women showed positive correlations with both (ρ = 0.446 and 0.657). SHBG correlated negatively with BCAA in both sexes (ρ = -0.452 and -0.571). In the BCAA group, no significant changes in sex hormones were observed after supplementation.

Conclusions: Cross-sectional associations between serum BCAA and testosterone levels in older adults with T2D were not observed longitudinally and may not reflect direct relationships, but rather influenced by insulin resistance or SHBG levels related to body composition. As this was an exploratory analysis with a small sample size, the results should be interpreted with caution.

## Introduction

Decreased serum sex hormone levels have been identified by the European Working Group on Sarcopenia in Older People (EWGSOP) as closely associated with age-related muscle volume and mass decline (i.e., sarcopenia) [1]. Nutritional intake has also been well-reported to modulate sarcopenia, particularly the nitrogen cycle [1], and serum branched-chain amino acids (BCAAs) levels correlate with body mass (BMI) and skeletal muscle indices [2].

Both sex hormones and BCAAs have anabolic effects on skeletal muscle [3,4], but the relationship between these anabolic factors and any potential sex differences is poorly studied. For example, studies in women with polycystic ovary syndrome and those in perimenopause report positive correlations between serum BCAA and testosterone levels [5,6]. In contrast, findings in men with testosterone deficiency are inconsistent, as some studies have shown increased BCAA levels [7], while others have reported decreased levels [8]. Although these observations suggest a link between amino acid metabolism and sex hormone levels, the cross-sectional relationship between the two has not been thoroughly investigated in populations at high risk for sarcopenia, such as postmenopausal women and older adults with type 2 diabetes (T2D) [9]. Moreover, while some studies have reported changes in serum amino acid profiles (e.g., cysteine, lysine, threonine, and tryptophan) following hormone replacement therapy [10-12], reports evaluating changes in sex hormone levels after amino acid supplementation are scarce. Thus, any causal relationships between total amino acid metabolism and subsequent sex hormone levels remain unclear.

Previously, we conducted a 24-week intervention study from 2019 to 2020 in older patients with T2D, comparing the effects of BCAA versus soy protein supplementation, and evaluated changes in serum amino acid concentrations [13]. To clarify the relationship between amino acid metabolism and sex hormone regulation in older adults with T2D, we investigated cross-sectional and longitudinal associations between serum amino acid concentrations and sex hormone levels using stored serum samples. The present exploratory post hoc study with a limited sample size includes sex-specific differences and changes in hormone concentrations following BCAA supplementation and may provide insights into the causal link between amino acid metabolism and sex hormone regulation.

## Materials and methods

Study design, setting, and participants

This study is a post hoc exploratory analysis based on data and stored serum samples from an interventional trial conducted between 2019 and 2020 involving older adults with T2D [13] and was conducted at the University of Tsukuba Hospital from February 27, 2024, to May 1, 2025. The original trial was registered at the University Hospital Medical Information Network Clinical Trials Registry (UMIN-CTR; UMIN000032368). Patients with T2D who regularly visited the University of Tsukuba Hospital were eligible if they were aged 65 to <80 years, had an HbA1c of 6.5-<8.5% at enrollment, and showed an HbA1c change ≤1.0% within the previous six months. Exclusion criteria were diabetes other than T2D, treatment with insulin, glucocorticoids, anabolic steroids, or growth hormone, estimated glomerular filtration rate (eGFR) <30 mL/min/1.73 m², proliferative diabetic retinopathy, inability to perform exercise due to musculoskeletal problems, or current treatment for malignancy. The original trial was a 24-week, prospective, randomized, open, blinded end-point (PROBE) study evaluating the effects of a daily supplement of 8 g of BCAAs (n = 21) versus 7.5 g of soy protein (n = 15) combined with physical activity training on skeletal muscle mass and HbA1c levels [13]. Participants were instructed to take the supplements after exercise or after dinner on days without exercise. The BCAA group received a total of 8 g of BCAAs, consisting of 4 g of leucine, 2 g of isoleucine, and 2 g of valine. Although the amino acid composition of the soy protein was not disclosed by the manufacturer, according to the Ministry of Education, Culture, Sports, Science and Technology (MEXT), 7.5 g of soy protein contains approximately 1.2 g of BCAAs (675 mg of leucine, 390 mg of isoleucine, and 113 mg of valine) [14]. Detailed inclusion criteria were described previously [13].

This study (approval number: R05-237, approved on February 27, 2024) and the previous study (approval number: H30-038, approved on May 18, 2018) [13] were conducted in accordance with the Declaration of Helsinki and were approved by the Ethics Committee of the University of Tsukuba Hospital, Tsukuba, Japan. In the previous study, all participants provided informed, written consent to use samples for this purpose [13].

Variables and measurements

The original study measured body composition (body weight, BMI, waist circumference, skeletal muscle mass, fat-free mass, and body fat mass), muscle strength (knee extension strength and handgrip strength), glucose metabolism (fasting plasma glucose, insulin, and homeostasis model assessment of insulin resistance (HOMA-IR)), renal function (serum creatinine, eGFR, and urinary albumin), serum amino acid concentrations, urinary parameters (urea nitrogen and creatinine), and food frequency questionnaire assessed by brief-type self-administered diet history questionnaire (BDHQ), which was developed and validated by Sasaki et al. [15]. Body composition was assessed using multi-frequency bioelectrical impedance analysis (InBody 720, Biospace, Tokyo, Japan). Knee extension strength (KES) was evaluated using isokinetic movement using a torque machine (Biodex System 3, Sakai Medical, Tokyo, Japan), and grip strength was measured with a Smedley analog grip strength meter (Toei Light, Saitama, Japan). Blood samples were collected in the early morning after overnight fasting. Serum samples for storage were collected in tubes containing clot activator and serum separator, allowed to clot for more than 60 minutes, centrifuged at 5,000 rpm for 15 minutes at 4°C, and stored at -80°C in aliquots. Serum amino acid concentrations were determined using liquid chromatography-mass spectrometry (LC-MS/MS) (LCMS-8030, Shimadzu, Kyoto, Japan), as described in the original report [13].

For the present study, we additionally measured total testosterone (TT), free testosterone (FT) measured by analog radioimmunoassay (aFT), sex hormone-binding globulin (SHBG), estradiol, luteinizing hormone (LH), follicle-stimulating hormone (FSH), and cortisol. TT was measured using the ARCHITECT Testosterone II assay (Abbott, Chiba, Japan); aFT was measured with a Free Testosterone RIA Kit SML (Immunotech s.r.o., Prague, Czech Republic); SHBG with the Human SHBG Quantikine enzyme-linked immunosorbent assay (ELISA) Kit (R&D Systems, Minneapolis, MN, USA); and estradiol, LH, FSH, and cortisol with the E Test Tosoh II (TOSOH, Tokyo, Japan). Calculated free testosterone (cFT) and bioavailable testosterone (bT) were estimated using the formula proposed by Vermeulen et al. [16]. Free androgen index (FAI) was calculated by dividing TT by SHBG then multiplying by 100, and androgen index (AI) was calculated by dividing aFT by TT.

Bias and sample size

Several factors were considered potential confounders, including BMI [2,17], waist circumference, body fat mass [17], insulin sensitivity [18], and skeletal muscle mass [2]. Due to the post hoc nature of this study, formal power calculations were not performed and the study was considered exploratory in nature.

Statistical analysis

Continuous variables were assessed for normality using the Shapiro-Wilk test. Normally distributed variables are expressed as mean (standard deviation), and non-normally distributed variables are expressed as median (interquartile range). Categorical variables are presented as n (%). For baseline characteristics, continuous variables with a normal distribution were compared using the t-test, while those without a normal distribution were analyzed using the Mann-Whitney U test. Categorical variables were compared using either Fisher’s exact test or the chi-squared test.

Analyses were performed primarily using a complete-case approach. Two participants (one male in the BCAA group and one female in the soy protein group) with cardiac pacemakers were excluded from body composition assessment because bioelectrical impedance analysis could not be performed. In addition, 12 participants (eight in the BCAA group (five men, three women) and four in the soy group (two men, two women)) without available cysteine measurements were excluded from analyses involving this amino acid.

Spearman’s rank correlation coefficients were used to evaluate the relationship between baseline amino acid concentrations and sex and adrenal cortex-related hormones (cross-sectional analysis), since some variables were not normally distributed. Given the small sample size, adjusted P-values could not be reliably calculated. As this analysis was exploratory in nature, multiple comparison adjustments were not performed, and the results should be interpreted with caution. We compared pre- and post-intervention levels of sex- and adrenal cortex-related hormones and amino acid concentrations by sex and intervention group. Paired t-tests were used for normally distributed data, and Wilcoxon signed-rank tests were applied for non-normally distributed data.

This was a post hoc exploratory analysis that was not pre-specified in the original study protocol. Statistical analysis was performed by IBM SPSS Statistics 29 (IBM Corp., Armonk, USA). P < 0.05 was considered statistically significant.

## Results

Baseline characteristics of study participants

Of the 36 participants in the original intervention study, data from 35 participants were included in the present analysis, as one was excluded due to the absence of stored post-intervention serum. Table 1 summarizes their baseline characteristics at the time of enrollment. The mean age ranged from 73 to 75 years, the median BMI ranged from 22.3 to 24.7 kg/m^2^, and the mean HbA1c ranged from 53 to 56 mmol/mol. Median HOMA-IR values in the BCAA and soy protein groups were 1.1 for men and 2.2 for women, respectively.

**Table 1:** Baseline characteristics of study participants. Data are presented as mean (standard deviation)ᵃ, n (%)ᵇ, or median (interquartile range)ᶜ. Variables expressed as ᵃ were analyzed using the t-test, those expressed as ᵇ using Fisher’s exact test or the chi-squared test, and those expressed as ᶜ using the Mann-Whitney U test. The P-value reflects the comparison between the overall male and female groups. The P-value for retinopathy was not calculated because there were no cases in either group. Total energy intake is based on the brief-type self-administered diet history questionnaire (BDHQ), and protein intake is based on 24-hour urine collection. BCAAs: branched-chain amino acids; eGFR: estimated glomerular filtration rate; HOMA-IR: homeostatic model assessment for insulin resistance.

Variables	Male			Female			P
	BCAA (n = 12)	Soy (n = 10)	Total (n = 22)	BCAA (n = 8)	Soy (n = 5)	Total (n = 13)	
Age^a^ (years)	73 (4)	73 (3)	73 (4)	75 (4)	74 (4)	75 (4)	0.162
Duration of diabetes^a^ (years)	20 (7)	22 (8)	21 (7)	22 (6)	18 (16)	21 (11)	0.960
Current smoking^b^	1 (8)	1 (10)	2 (9)	0 (0)	0 (0)	0 (0)	0.519
Alcohol consumption^c^ (g/day)	2 (0, 23)	0 (0, 11)	0 (0, 21)	0 (0, 0)	0 (0, 0)	0 (0, 0)	0.091
Total energy^a^ (kcal/day)	2,136 (439)	1,931 (512)	2,042 (473)	1,969 (446)	1,550 (575)	1,808 (521)	0.181
Protein intake^c^ (g/day)	73 (59, 84)	70 (58, 80)	73 (59, 80)	49 (40, 58)	57 (46, 76)	55 (42, 62)	0.008
Protein intake by body weight^a^ (g/kg/day)	1.1 (0.2)	1.1 (0.2)	1.1 (0.2)	1.0 (0.3)	1.2 (0.4)	1.1 (0.4)	0.707
Diabetic complications							
Neuropathy^b^	4 (33)	2 (20)	6 (27)	0 (0)	0 (0)	0 (0)	0.064
Retinopathy^b^	0 (0)	0 (0)	0 (0)	0 (0)	0 (0)	0 (0)	—
Nephropathy^b^	3 (25)	5 (50)	8 (36)	4 (50)	0 (0)	4 (31)	1.000
Cardiovascular disease^b^	3 (25)	2 (20)	5 (23)	1 (13)	3 (60)	4 (31)	0.698
Body mass index^c^ (kg/m^2^)	24.0 (22.4, 25.3)	22.3 (20.6, 24.8)	23.5 (21.5, 25.1)	24.7 (20.1, 26.4)	23.4 (21.2, 23.7)	23.6 (21.2, 25.1)	0.853
Skeletal muscle mass^a^ (kg)	27.9 (3.0)	25.5 (3.3)	26.8 (3.3)	19.5 (3.3)	18.0 (1.9)	19.0 (2.9)	<0.001
Skeletal muscle mass index^c^ (kg/m^2^)	7.7 (7.0, 8.1)	6.9 (6.4, 7.8)	7.5 (6.7, 7.8)	5.9 (5.3, 7.1)	5.8 (5.0, 6.0)	5.9 (5.3, 6.1)	<0.001
Fat free mass^a^ (kg)	50.6 (5.3)	46.9 (5.6)	48.8 (5.6)	36.7 (5.6)	34.1 (2.8)	35.8 (4.9)	<0.001
Body fat mass^a^ (kg)	17.5 (6.5)	14.6 (4.3)	16.1 (5.7)	19.4 (8.2)	16.3 (3.5)	18.4 (7.0)	0.309
Waist circumference^a^ (cm)	88 (7)	84 (7)	86 (7)	88 (12)	84 (4)	87 (9)	0.861
Grip strength^c^ (kg)	37 (30, 44)	36 (31, 40)	36 (31, 41)	22 (19, 24)	22 (19, 27)	22 (19, 25)	<0.001
eGFR^a^ (mL/min/1.73 m^2^)	68 (13)	63 (11)	66 (12)	76 (16)	72 (23)	75 (18)	0.093
HbA1c^a^ (mmol/mol)	56 (7)	53 (3)	55 (6)	55 (6)	53 (3)	55 (5)	0.792
Fasting plasma glucose^a^ (mmol/L)	7.9 (1.5)	8.0 (1.2)	7.9 (1.4)	7.3 (1.4)	8.3 (1.2)	7.7 (1.4)	0.665
HOMA-IR^c^	1.2 (1.0, 1.9)	0.9 (0.7, 1.5)	1.1 (0.7, 1.6)	2.8 (1.9, 5.0)	2.2 (1.7, 3.6)	2.2 (1.2, 2.8)	0.012

Table 2 presents baseline levels of sex- and adrenal cortex-related hormones and serum amino acid levels by intervention group and sex. Estradiol levels were above the assay's lower detection limit (20 pg/mL) in only three of the 35 cases, and thus, the data are not shown. TT, aFT, cFT, bT, FAI, and AI were higher in men than in women, whereas LH and FSH were higher in women. Blood concentrations of BCAAs (except valine), total amino acids, essential amino acids (EAAs), and non-essential amino acids (NEAAs) were also generally higher in men than in women. The other baseline serum amino acids are shown in Table 3.

**Table 2:** Baseline sex- and adrenal cortex-related hormones and serum amino acids levels before the intervention. Data are expressed as the mean (standard deviation) or median (interquartile range). Normally distributed variables were compared using the t-test, and non-normally distributed variables were compared using the Mann-Whitney U test. The P-value reflects the comparison between the overall male and female groups. AI: androgen index; aFT: free testosterone measured by analog radioimmunoassay; BCAA: branched-chain amino acid; bT: bioavailable testosterone; cFT: calculated free testosterone; EAA: essential amino acid; FAI: free androgen index; FSH: follicle-stimulating hormone; LH: luteinizing hormone; NEAA: non-essential amino acid; SHBG: sex hormone-binding globulin; TT: total testosterone.

Variables	Male			Female			P
	BCAA (n = 12)	Soy (n = 10)	Total (n = 22)	BCAA (n = 8)	Soy (n = 5)	Total (n = 13)	
Sex and adrenal cortex-related hormones							
TT (nmol/L)	17.5 (14.3, 22.9)	21.0 (18.5, 23.3)	19.1 (16.8, 22.1)	1.0 (0.7, 1.3)	1.1 (0.9, 1.2)	1.1 (0.8, 1.3)	<0.001
aFT (pmol/L)	24.4 (19.2, 31.1)	23.7 (19.0, 27.1)	24.1 (19.0, 29.0)	2.1 (1.5, 3.3)	1.7 (1.2, 4.0)	1.7 (1.4, 3.5)	<0.001
cFT (pmol/L)	0.37 (0.29, 0.47)	0.40 (0.26, 0.51)	0.38 (0.27, 0.49)	0.01 (0.01, 0.02)	0.01 (0.01, 0.03)	0.01 (0.01, 0.02)	<0.001
bT (nmol/L)	8.96 (6.77, 11.26)	8.63 (5.95, 11.78)	8.96 (6.47, 11.26)	0.32 (0.21, 0.54)	0.25 (0.22, 0.82)	0.26 (0.21, 0.55)	<0.001
LH (IU/L)	8 (8, 14)	10 (6, 15)	9 (7, 13)	20 (14, 38)	20 (14, 27)	20 (14, 27)	<0.001
FSH (IU/L)	31 (23, 70)	20 (16, 54)	29 (18, 56)	73 (60, 110)	74 (47, 97)	74 (56, 99)	<0.001
Cortisol (nmol/L)	373 (74)	416 (110)	393 (92)	330 (88)	572 (182)	423 (175)	0.508
SHBG (nmol/L)	29 (23, 39)	45 (32, 67)	36 (27, 51)	50 (36, 55)	50 (14, 87)	50 (28, 63)	0.243
FAI	57.1 (42.2, 81.4)	55.9 (31.9, 64.6)	57.1 (39.6, 67.8)	1.9 (1.3, 3.7)	1.5 (1.2, 8.9)	1.5 (1.3, 4.4)	<0.001
AI	1.3 (1.3, 1.6)	1.2 (0.9, 1.5)	1.3 (1.1, 1.6)	2.1 (1.8, 2.8)	1.8 (1.3, 3.2)	1.9 (1.7, 2.8)	<0.001
Amino acids							
BCAAs (µmol/L)	442 (64)	403 (43)	424 (58)	354 (58)	368 (90)	359 (69)	0.005
Valine (µmol/L)	223 (36)	200 (20)	213 (31)	188 (26)	194 (42)	190 (31)	0.053
Isoleucine (µmol/L)	82 (11)	75 (8)	79 (10)	63 (13)	65 (21)	64 (16)	0.002
Leucine (µmol/L)	137 (19)	128 (18)	133 (19)	103 (21)	109 (29)	105 (23)	<0.001
Total amino acids (µmol/L)	3,066 (214)	2,964 (270)	3,019 (240)	2,911 (310)	2,725 (195)	2,839 (279)	0.052
EAAs (µmol/L)	896 (92)	834 (74)	868 (88)	757 (85)	770 (116)	762 (93)	0.002
NEAAs (µmol/L)	2,170 (157)	2,130 (211)	2,152 (180)	2,154 (241)	1,955 (119)	2,078 (221)	0.287

**Table 3:** Baseline serum amino acid levels other than BCAAs of study participants before the intervention. Data are expressed as the mean (standard deviation) or median (interquartile range). Normally distributed variables were compared using the t-test, and non-normally distributed variables were compared using the Mann-Whitney U test. The P-value reflects the comparison between the overall male and female groups. Amino acid concentrations other than BCAAs (valine, isoleucine, leucine), total amino acids, EAAs, and NEAAs are presented. BCAA: branched-chain amino acid; EAA: essential amino acid; NEAA: non-essential amino acid.

Variables	Male			Female			P
	BCAA (n = 12)	Soy (n = 10)	Total (n = 22)	BCAA (n = 8)	Soy (n = 5)	Total (n = 13)	
EAAs							
Tryptophan (µmol/L)	51 (6)	50 (11)	50 (9)	48 (10)	51 (10)	49 (10)	0.740
Threonine (µmol/L)	136 (19)	139 (23)	138 (21)	119 (22)	121 (24)	120 (21)	0.022
Lysine (µmol/L)	125 (20)	109 (21)	118 (21)	108 (8)	109 (19)	108 (13)	0.153
Histidine (µmol/L)	56 (8)	53 (7)	54 (7)	50 (10)	47 (4)	49 (8)	0.046
Methionine (µmol/L)	26 (3)	25 (3)	25 (3)	24 (4)	20 (2)	22 (4)	0.009
Phenylalanine (µmol/L)	60 (7)	56 (9)	58 (8)	54 (13)	54 (14)	54 (13)	0.238
NEAAs							
Tyrosine (µmol/L)	59 (9)	59 (10)	59 (9)	63 (12)	63 (9)	63 (11)	0.198
Cystine (µmol/L)	8.7 (5.9, 10.9)	7.9 (5.9, 9.9)	8.0 (5.9, 10.6)	8.5 (6.8, 11.9)	4.5 (3.4, 19.1)	7.4 (4.1, 12.7)	0.960
Asparagine (µmol/L)	75 (6)	74 (8)	75 (7)	69 (8)	66 (7)	68 (8)	0.008
Aspartic acid (µmol/L)	38 (8)	35 (4)	37 (7)	36 (6)	38 (7)	36 (6)	0.919
Serine (µmol/L)	197 (37)	193 (23)	195 (31)	193 (35)	187 (18)	191 (29)	0.673
Alanine (µmol/L)	300 (49)	294 (62)	297 (54)	301 (49)	296 (69)	299 (55)	0.922
Glycine (µmol/L)	202 (22)	208 (38)	205 (30)	219 (35)	192 (17)	208 (32)	0.737
Glutamine (µmol/L)	1,063 (102)	1,017 (134)	1,042 (117)	1,059 (128)	922 (65)	1,007 (125)	0.403
Cysteine (µmol/L)	0.9 (0.6)	1.2 (0.4)	1.1 (0.5)	0.7 (0.4)	0.9 (0.7)	0.8 (0.5)	0.229
Glutamic acid (µmol/L)	54 (14)	56 (15)	55 (14)	51 (15)	47 (15)	50 (14)	0.297
Proline (µmol/L)	85 (78, 102)	95 (77, 116)	89 (77, 105)	76 (69, 86)	71 (58, 90)	75 (68, 85)	0.012
Arginine (µmol/L)	82 (16)	88 (13)	85 (15)	72 (14)	61 (13)	67 (14)	0.002

Cross-sectional relationship between sex- and adrenal cortex-related hormone levels and serum BCAA concentrations

**Figure 1:**
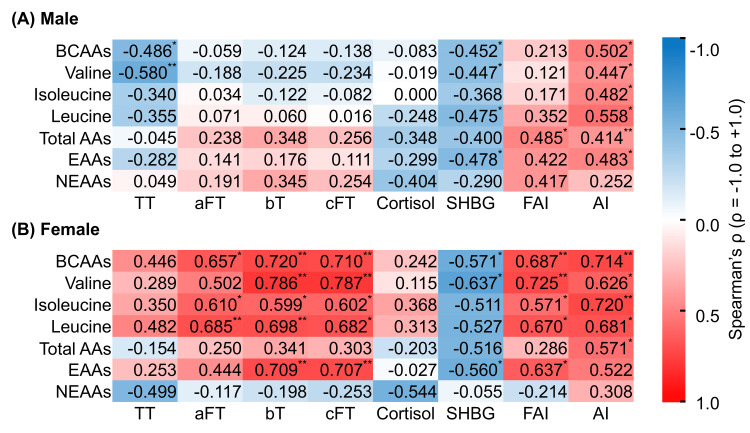
Heatmap of the cross-sectional correlation matrix of baseline serum amino acid concentrations, including BCAAs with sex and adrenal cortex-related hormone levels. (A) Male, (B) Female. Data are presented as Spearman’s correlation coefficients. In the heatmap, colors represent the strength and direction of Spearman’s correlation coefficients (ρ): white corresponds to ρ = 0, blue to ρ = -1, and red to ρ = 1. Asterisks indicate statistical significance: *P < 0.05; **P < 0.01. AA: amino acid; AI: androgen index; aFT: free testosterone measured by analog radioimmunoassay; BCAA: branched-chain amino acid; bT: bioavailable testosterone; cFT: calculated free testosterone; EAA: essential amino acid; FAI: free androgen index; NEAA: non-essential amino acid; SHBG: sex hormone-binding globulin; TT: total testosterone.

In the baseline cross-sectional data of men, serum BCAA concentrations were significantly correlated with TT and SHBG and positively correlated with AI (P < 0.05), whereas no significant associations were found with free testosterone indices, including aFT, bT, or cFT (Figures 1A, 1B).

**Figure 2:**
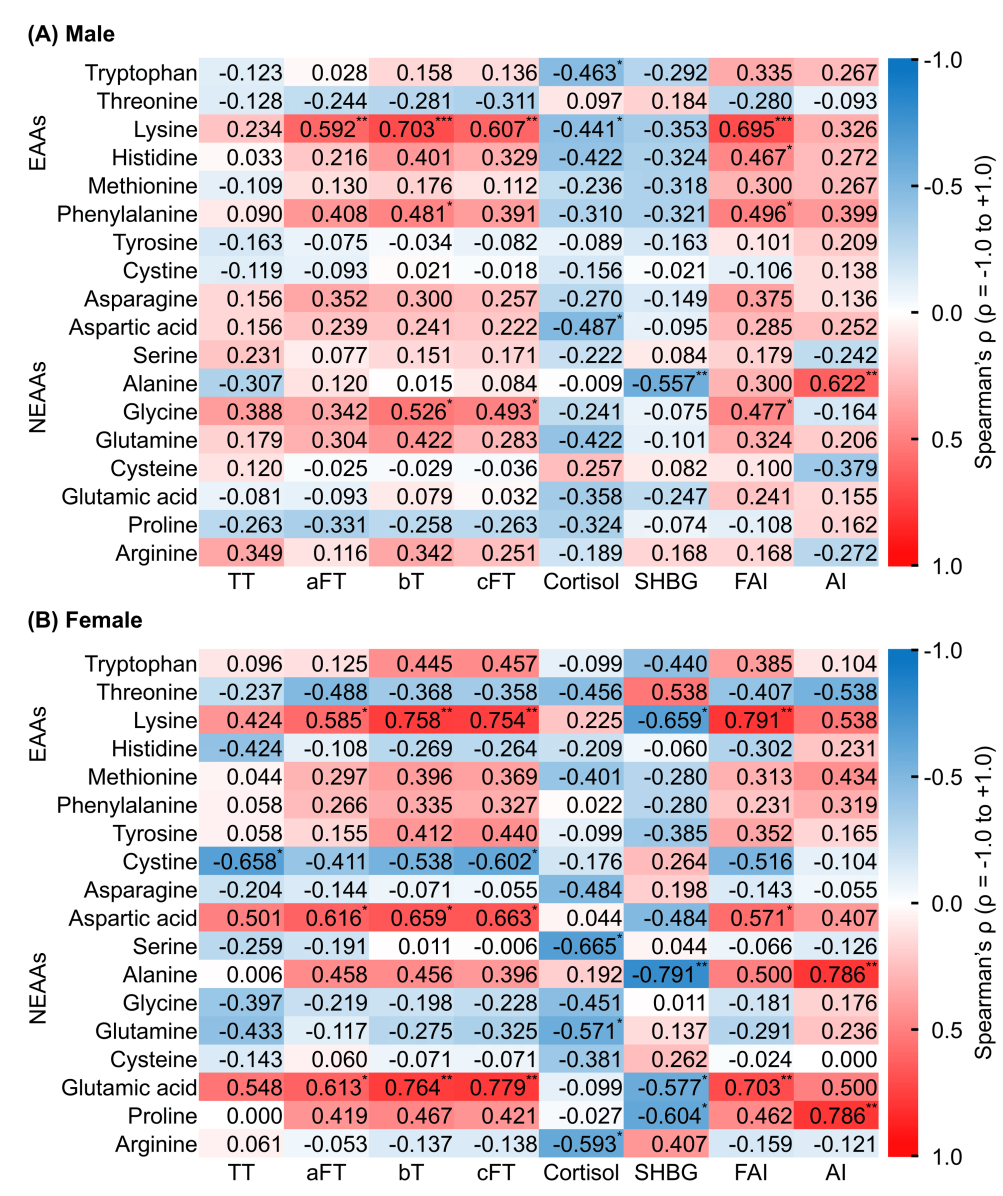
Heatmap of the cross-sectional correlation matrix of baseline blood amino acid concentrations other than BCAAs with sex- and adrenal cortex-related hormone levels. (A) Male, (B) Female. Data are presented as Spearman’s correlation coefficients. In the heatmap, colors represent the strength and direction of Spearman’s correlation coefficients (ρ): white corresponds to ρ = 0, blue to ρ = -1, and red to ρ = 1. Asterisks indicate statistical significance: *P < 0.05; **P < 0.01; ***P < 0.001. AI: androgen index; aFT: free testosterone measured by analog radioimmunoassay; BCAA: branched-chain amino acid; bT: bioavailable testosterone; cFT: calculated free testosterone; EAA: essential amino acid; FAI: free androgen index; NEAA: non-essential amino acid; SHBG: sex hormone-binding globulin; TT: total testosterone.

On the other hand, women showed significant positive correlations between serum BCAA concentrations and aFT, bT, cFT, and AI, plus significant negative correlations with SHBG (P < 0.05). Although the positive correlation with TT did not reach statistical significance (P = 0.126), the correlation was moderate in strength (Spearman’s ρ = 0.446). Figures 2A, 2B show the associations between non-BCAA amino acid levels and sex- and adrenal cortex-related hormone levels.

Changes in sex- and adrenal cortex-related hormones and blood amino acid levels in response to nutritional intervention

In the BCAA group (n = 20), sex- and adrenal cortex-related hormone levels did not change significantly in either sex, except for a decrease in FSH levels in men (Table 4). Similarly, in the soy protein group (n = 15), neither sex exhibited significant hormonal changes. Serum amino acid concentrations showed significant increases in BCAAs, total amino acids, EAAs, and NEAAs in women in the BCAA group, whereas no significant changes were observed in men in the BCAA group or in either sex in the soy protein group. There were no significant changes in body composition parameters in either group. Changes in individual amino acid concentrations other than BCAAs before and after the intervention are summarized in Table 5.

**Table 4:** Pre- and post-intervention changes in sex- and adrenal cortex-related hormones, serum amino acid levels, and body composition by intervention group and sex. Data are expressed as the mean (standard deviation) or median (interquartile range). P-values represent the results of pre- and post-intervention comparisons within each group and sex. Normally distributed variables were analyzed using the paired t-test, and non-normally distributed variables were analyzed using the Wilcoxon signed-rank test. Baseline values in this table are the same as those presented in Table 2. AI: androgen index; aFT: free testosterone measured by analog radioimmunoassay; BCAA: branched-chain amino acid; bT: bioavailable testosterone; cFT: calculated free testosterone; EAA: essential amino acid; FAI: free androgen index; FSH: follicle-stimulating hormone; LH: luteinizing hormone; NEAA: non-essential amino acid; SHBG: sex hormone-binding globulin; TT: total testosterone.

Variables	Baseline	24 Weeks	P	Baseline	24 Weeks	P
BCAA Group (n = 20)	Male (n = 12)			Female (n = 8)		
Sex- and adrenal cortex-related hormones						
TT (nmol/L)	17.5 (14.3, 22.9)	17.5 (14.1, 20.4)	0.875	1.0 (0.7, 1.3)	0.8 (0.7, 1.3)	0.671
aFT (pmol/L)	24.4 (19.2, 31.1)	25.7 (18.2, 31.6)	0.638	2.1 (1.5, 3.3)	1.4 (1.4, 3.1)	0.336
cFT (pmol/L)	0.37 (0.29, 0.47)	0.37 (0.29, 0.51)	1.000	0.01 (0.01, 0.02)	0.01 (0.01, 0.02)	0.889
bT (nmol/L)	8.96 (6.77, 11.26)	9.01 (6.51, 11.26)	0.937	0.32 (0.21, 0.54)	0.25 (0.18, 0.55)	0.752
LH (IU/L)	8 (8, 14)	9 (7, 15)	0.875	20 (14, 38)	20 (14, 40)	0.497
FSH (IU/L)	31 (23, 70)	32 (24, 77)	0.018	73 (60, 110)	75 (58, 121)	0.484
Cortisol (nmol/L)	373 (74)	386 (93)	0.611	330 (88)	306 (64)	0.425
SHBG (nmol/L)	29 (23, 39)	34 (23, 41)	0.530	50 (36, 55)	36 (34, 64)	0.401
FAI	57.1 (42.2, 81.4)	56.9 (39.5, 85.5)	0.875	1.9 (1.3, 3.7)	1.9 (1.1, 3.4)	1.000
AI	1.4 (1.3, 1.6)	1.3 (1.3, 1.6)	0.583	2.1 (1.8, 2.8)	1.9 (1.7, 2.6)	0.263
Amino acids						
BCAAs (µmol/L)	442 (64)	462 (68)	0.378	354 (58)	422 (64)	<0.001
Valine (µmol/L)	223 (36)	237 (35)	0.289	188 (26)	226 (30)	0.003
Isoleucine (µmol/L)	82 (11)	83 (15)	0.781	63 (13)	71 (17)	0.008
Leucine (µmol/L)	137 (19)	142 (22)	0.457	103 (21)	125 (22)	<0.001
Total amino acids (µmol/L)	3,066 (214)	3,116 (171)	0.346	2,911 (310)	3,198 (235)	0.008
EAAs (µmol/L)	896 (92)	902 (89)	0.836	757 (85)	875 (87)	0.001
NEAAs (µmol/L)	2,170 (157)	2,214 (140)	0.336	2,154 (241)	2,323 (161)	0.030
Body composition						
Body mass index (kg/m^2^)	24.0 (22.4, 25.3)	23.8 (22.4, 24.6)	0.209	24.7 (20.1, 26.4)	24.3 (20.3, 26.2)	0.263
Skeletal muscle mass (kg)	27.9 (3.0)	27.8 (3.1)	0.697	19.5 (3.3)	19.5 (3.2)	0.826
Skeletal muscle mass index (kg/m^2^)	7.7 (7.0, 8.1)	7.4 (7.0, 8.0)	0.722	5.9 (5.3, 7.1)	6.1 (5.3, 7.0)	0.779
Fat free mass (kg)	50.6 (5.3)	50.5 (5.3)	0.847	36.7 (5.6)	36.7 (5.5)	0.900
Body fat mass (kg)	17.5 (6.5)	17.1 (6.2)	0.289	19.4 (8.2)	19.0 (7.7)	0.190
Waist circumference (cm)	88 (7)	88 (7)	0.335	88 (12)	88 (11)	0.107
Soy protein group (n = 15)	Male (n = 10)			Female (n = 5)		
Sex- and adrenal cortex-related hormones						
TT (nmol/L)	21.0 (18.5, 23.3)	21.0 (17.4, 23.9)	0.878	1.1 (0.9, 1.2)	0.9 (0.7, 1.5)	0.068
aFT (pmol/L)	23.7 (19.0, 27.1)	27.7 (19.4, 30.3)	0.202	1.2 (0.0, 1.7)	1.4 (0.0, 1.7)	0.593
cFT (pmol/L)	0.40 (0.26, 0.51)	0.43 (0.22, 0.45)	0.169	0.01 (0.01, 0.03)	0.01 (0.00, 0.01)	0.144
bT (nmol/L)	8.63 (5.95, 11.78)	9.60 (5.18, 10.69)	0.203	0.25 (0.22, 0.82)	0.29 (0.15, 0.93)	0.144
LH (IU/L)	10 (6, 15)	9 (7, 15)	0.475	19 (14, 28)	19 (12, 27)	0.144
FSH (IU/L)	20 (16, 54)	20 (16, 54)	0.093	80 (49, 101)	79 (49, 107)	0.465
Cortisol (nmol/L)	416 (110)	441 (107)	0.454	532 (184)	407 (106)	0.362
SHBG (nmol/L)	45 (32, 67)	46 (34, 59)	0.799	50 (14, 87)	52 (15, 88)	1.000
FAI	55.9 (31.9, 64.6)	50.4 (30.6, 59.8)	0.285	1.5 (1.2, 8.9)	1.8 (0.8, 9.5)	0.273
AI	1.2 (0.9, 1.5)	1.2 (1.0, 1.6)	0.386	1.8 (1.3, 3.2)	2.3 (1.7, 3.1)	0.465
Amino acids						
BCAAs (µmol/L)	403 (43)	428 (49)	0.266	331 (42)	336 (73)	0.488
Valine (µmol/L)	200 (20)	216 (24)	0.066	194 (42)	197 (53)	0.666
Isoleucine (µmol/L)	75 (8)	81 (13)	0.067	65 (21)	69 (25)	0.415
Leucine (µmol/L)	128 (18)	131 (13)	0.379	109 (29)	112 (37)	0.452
Total amino acids (µmol/L)	2,964 (270)	3,046 (113)	0.370	2,675 (184)	2,688 (108)	0.394
EAAs (µmol/L)	834 (74)	880 (58)	0.222	726 (70)	729 (80)	0.588
NEAAs (µmol/L)	2,130 (211)	2,166 (99)	0.557	1,949 (137)	1,959 (45)	0.456
Body composition						
Body mass index (kg/m^2^)	22.3 (20.6, 24.8)	22.6 (20.5, 24.8)	0.185	23.4 (21.2, 23.7)	22.9 (21.3, 23.6)	0.893
Skeletal muscle mass (kg)	25.5 (3.3)	25.5 (3.3)	0.653	18.0 (1.9)	18.2 (2.0)	0.287
Skeletal muscle mass index (kg/m^2^)	6.9 (6.4, 7.8)	7.0 (6.4, 7.7)	0.859	5.8 (5.0, 6.0)	5.9 (5.0, 6.2)	0.144
Fat free mass (kg)	46.9 (5.6)	46.7 (5.5)	0.640	34.1 (2.9)	34.6 (3.1)	0.262
Body fat mass (kg)	14.6 (4.3)	14.9 (4.5)	0.368	16.3 (3.5)	16.0 (3.2)	0.678
Waist circumference (cm)	84 (7)	84 (7)	0.335	84 (4)	83 (4)	0.404

**Table 5:** Pre- and post-intervention changes in serum amino acid levels other than BCAAs by intervention group and sex. Data are expressed as the mean (standard deviation) or median (interquartile range). P-values represent the results of pre- and post-intervention comparisons within each group and sex. Normally distributed variables were analyzed using the paired t-test, and non-normally distributed variables were analyzed using the Wilcoxon signed-rank test. Baseline values in this table are the same as those presented in Table 3. BCAA: branched-chain amino acid.

Variables	Baseline	24 Weeks	P	Baseline	24 Weeks	P
BCAA group (n = 20)	Male (n = 12)			Female (n = 8)		
Tryptophan (µmol/L)	51 (6)	54 (7)	0.160	48 (10)	56 (11)	0.006
Threonine (µmol/L)	136 (19)	126 (22)	0.053	119 (22)	131 (38)	0.150
Lysine (µmol/L)	125 (20)	121 (18)	0.053	108 (8)	121 (9)	0.002
Histidine (µmol/L)	56 (8)	52 (5)	0.105	50 (10)	54 (9)	0.041
Methionine (µmol/L)	26 (3)	26 (4)	0.712	24 (4)	27 (4)	0.048
Phenylalanine (µmol/L)	60 (7)	60 (7)	0.707	54 (13)	63 (12)	0.004
Tyrosine (µmol/L)	59 (9)	60 (11)	0.539	63 (12)	73 (13)	0.006
Cystine (µmol/L)	8.7 (5.9, 10.9)	8.1 (5.4, 12.4)	0.388	8.5(6.8, 11.9)	5.1 (4.2, 7.3)	0.263
Asparagine (µmol/L)	75 (6)	78 (6)	0.296	69 (8)	75 (13)	0.088
Aspartic acid (µmol/L)	38 (8)	37 (6)	0.651	36 (6)	40 (8)	0.219
Serine (µmol/L)	197 (37)	196 (23)	0.841	193 (35)	213 (48)	0.036
Alanine (µmol/L)	300 (49)	314 (40)	0.198	301 (49)	322 (51)	0.287
Glycine (µmol/L)	202 (22)	196 (26)	0.366	219 (35)	223 (27)	0.805
Glutamine (µmol/L)	1,063 (102)	1,093 (107)	0.285	1,059 (128)	1,147 (79)	0.032
Cysteine (µmol/L)	1.0 (0.6)	1.0 (0.4)	0.950	0.8 (0.4)	0.7 (0.5)	0.335
Glutamic acid (µmol/L)	54 (14)	52 (11)	0.642	51 (15)	57 (11)	0.344
Proline (µmol/L)	85 (78, 102)	91 (78, 108)	0.814	76 (69, 86)	86 (76, 102)	0.263
Arginine (µmol/L)	82 (16)	85 (20)	0.477	72 (14)	78 (15)	0.156
Soy group (n = 15)	Male (n = 10)			Female (n = 5)		
Tryptophan (µmol/L)	50 (11)	52 (7)	0.155	51 (10)	51 (9)	0.979
Threonine (µmol/L)	139 (23)	146 (14)	0.103	121 (24)	121 (25)	0.979
Lysine (µmol/L)	109 (21)	117 (12)	0.098	109 (19)	106 (15)	0.428
Histidine (µmol/L)	53 (7)	53 (7)	0.258	47 (4)	47 (4)	0.570
Methionine (µmol/L)	25 (3)	25 (3)	0.420	20 (2)	21 (3)	0.534
Phenylalanine (µmol/L)	56 (9)	56 (5)	0.400	54 (14)	56 (17)	0.546
Tyrosine (µmol/L)	59 (10)	61 (8)	0.233	63 (9)	64 (10)	0.488
Cystine (µmol/L)	7.9 (5.9, 9.9)	7.2 (4.3, 15.8)	0.386	4.5 (3.4, 19.1)	7.5 (5.0, 9.5)	0.686
Asparagine (µmol/L)	74 (8)	78 (9)	0.107	66 (7)	67 (5)	0.875
Aspartic acid (µmol/L)	35 (4)	36 (8)	0.351	38 (7)	35 (7)	0.448
Serine (µmol/L)	193 (23)	192 (24)	0.489	187 (18)	191 (5)	0.590
Alanine (µmol/L)	294 (62)	299 (66)	0.266	296 (69)	292 (77)	0.806
Glycine (µmol/L)	208 (38)	214 (30)	0.250	192 (17)	198 (38)	0.642
Glutamine (µmol/L)	1,017 (134)	1,043 (88)	0.218	922 (65)	968 (62)	0.457
Cysteine (µmol/L)	1.1 (0.4)	1.0 (0.3)	0.261	0.9 (0.7)	0.8 (0.3)	0.784
Glutamic acid (µmol/L)	56 (15)	48 (20)	0.091	47 (15)	46 (12)	0.928
Proline (µmol/L)	95 (77, 116)	82 (77, 113)	0.285	71 (58, 90)	74 (56, 80)	0.500
Arginine (µmol/L)	88 (13)	89 (12)	0.355	61 (13)	68 (13)	0.076

## Discussion

This study demonstrated a cross-sectional positive correlation between serum BCAA levels and TT or FT in women and a negative correlation with TT in men, indicating that the association with TT differed by sex. However, in the longitudinal analysis of the BCAA and soy protein groups, sex- and adrenal cortex-related hormone levels did not change significantly in either sex. These results suggest that there is no causal relationship between serum BCAA levels and sex hormones. To the best of our knowledge, this is the first exploratory post hoc study to provide preliminary findings supporting the association between amino acid metabolism and sex hormones, both cross-sectionally and longitudinally, in elderly individuals with T2D.

Association between BCAA and sex hormones in women

In the cross-sectional analysis, serum BCAA concentrations were positively associated with TT and FT in women. This finding is consistent with previous reports of polycystic ovary syndrome (PCOS) and premenopausal women, which demonstrated positive correlations between BCAA and testosterone [5,6]. Furthermore, testosterone levels are linked to maintenance of blood glucose, insulin resistance, and body fat mass [19], while insulin resistance may promote testosterone secretion from the ovaries [20]. In addition, testosterone is continually released by the ovaries and adrenal cortex, even after menopause [21]. Since BCAA concentrations are positively associated with body composition [2] and insulin resistance [22,23], these combined factors may contribute to the reported positive association between BCAA and testosterone in women. Furthermore, hyperinsulinemia stemming from insulin resistance, which is strongly related to BCAA concentrations [22,23], is known to suppress SHBG production [24], and, in the present study, SHBG was negatively associated with BCAA and AI (FT/TT) was positively associated with BCAA. The positive association between AI and BCAA in women may be explained by decreased SHBG with higher BCAA levels, resulting in a relative increase in FT. Although albumin may also influence the free fraction of testosterone, previous studies have reported that, at physiological serum concentrations within the range of 40-50 g/L, its impact on FT is minimal [25]. Indeed, the FT indices (aFT, bT, and cFT) were also positively associated with BCAA in women, consistent with this interpretation, and this finding is in line with previous reports that FAI is positively associated with BCAA [5].

Association between BCAA and sex hormones in men

In contrast, serum BCAA concentrations were negatively associated with TT (but not FT) in men. This is the first study to report this relationship in older men with T2D. In men, testosterone levels are known to be negatively associated with body composition and insulin resistance [19], whereas BCAA levels are positively associated with these factors [2,22,23]. Thus, body composition and insulin resistance may contribute to the observed negative association between BCAA and testosterone in men. Indeed, in men with hypogonadism and insulin resistance, serum BCAA and several amino acids were reported to be significantly reduced compared with men without insulin resistance [8], consistent with the present findings.

Moreover, although aFT, bT, and cFT were not significantly associated with BCAA in men, AI was positively associated with BCAA. This may be explained by the fact that TT, which is the denominator of AI, was negatively associated with BCAA. As a result, fluctuations in TT may have been directly reflected in AI and could account for the observed positive association between AI and BCAA.

Longitudinal analysis and interpretation

In the longitudinal analysis, women in the BCAA group showed a significant increase in serum BCAA concentrations. However, no significant changes were observed in sex hormone levels. This study is meaningful in that the intervention allowed us to examine whether changes in serum BCAA concentrations would affect sex hormones, thereby assessing causality. Such causal relationships cannot be clarified by cross-sectional analysis alone. However, as serum BCAA concentrations did not significantly increase in men and evaluation of causality was not possible, the observed differences may reflect sex-related variations in response to amino acid supplementation. Compared to men and older adults, women and younger individuals are more likely to exhibit a positive nitrogen balance following protein intake [26]. After consuming the same amount of protein, women have been reported to show higher area under the curve (AUC) values for total amino acids, EAAs, and leucine [27].

Furthermore, in previous studies in which testosterone was supplemented in postmenopausal women and transgender patients, serum concentrations of BCAA and many other amino acids showed little change [10-12]. Taken together with the results in women from the present study, in which BCAA concentrations changed but testosterone levels did not, our collective results suggest little bidirectional, causal influence between BCAA and testosterone. These results also suggest that, although BCAA and testosterone share anabolic effects on skeletal muscle [3,4] and their circulating levels may be regulated by common biological backgrounds, they do not influence each other directly and may act independently. These findings provide a novel perspective for interventions aimed at preventing sarcopenia in older adults.

The effect of isoflavones on testosterone levels

In the previous study, 7.5 g of soy protein per day was administered to the soy protein group [13] and regular soy flour contains approximately 211-321 mg of soy isoflavones per 100 g [28]. Based on this, the estimated isoflavone content of the supplement used in the present study is approximately 16-24 mg per day. Soy isoflavones are known binders to estrogen receptors and thus may influence levels of SHBG and testosterone [29]. However, in this study, no significant changes were observed in testosterone, LH, FSH, or SHBG levels in the soy protein group before or after the intervention. Similarly, previous studies administering 20-50 mg/day of isoflavones to postmenopausal women reported no significant changes in estradiol, testosterone, or gonadotropin levels [30], consistent with our study. Based on these results, the modulatory effect of soy protein isoflavones is considered minimal.

Limitations

This study has several limitations. First, the sample size was small (n = 35), and only 13 women were included. Therefore, cross-sectional associations observed in women should be interpreted with caution, as they may reflect chance findings in this limited sample. Because it was an exploratory post hoc study, we were unable to perform fully adjusted analyses for potential confounders and the study was subject to statistical constraints. Based on previous literature, insulin resistance and SHBG, both influenced by body composition, may have acted as confounders in the relationship between serum BCAA concentrations and testosterone levels; however, unfortunately, mediation analysis could not be conducted due to the limited sample size and this point is reserved for future research. Second, in the longitudinal analysis, men in the BCAA supplementation group did not show changes in serum BCAA concentrations. Therefore, the causal relationship between BCAA concentrations and testosterone could not be adequately clarified in men. The dosage of BCAA supplementation was determined based on previous studies as a dose that could potentially promote skeletal muscle anabolism, but it may have been insufficient to consistently alter circulating BCAA levels. Moreover, BCAA metabolism is influenced by insulin resistance, and differences in HOMA-IR between men and women in the present study may also have contributed to the observed sex differences in BCAA concentration changes. Third, as the study cohort was limited to older adults with T2D, the generalizability of our results to differing populations, such as older adults without T2D, is limited.

## Conclusions

In this exploratory post hoc analysis of older adults with T2D, cross-sectional associations were observed between serum BCAA concentrations and testosterone, showing a positive association in women and a negative association in men. However, since BCAA supplementation did not alter testosterone levels, the cross-sectional association may not represent a direct relationship. It may instead be explained by confounding factors such as body composition, insulin resistance, and/or SHBG. As this was an exploratory post hoc analysis, the findings should be interpreted with caution, and future studies with larger sample sizes and interventional designs are needed to confirm that this association excludes a causal relationship.
